# General Pathology, or the Science of the Causes, Nature and Course of the Processes of Disease

**Published:** 1904-04

**Authors:** 


General Pathology, or the Science of the Causes, Nature and Course of the Processes of Disease. By Dr. Ernest Ziegler, Professor of Pathological Anatomy and of General Pathology in the University of Freiburg in Breisgau. Translated from the tenth revised German edition, and edited by Alfred Scott Warthin, M. D., Professor of Pathology and Director of the Pathological Laboratory in the University of Michigan, Ann Arbor, Mich. Octavo, pp. 783. Illustrated. New York: William Wood & Co. 1903. (Cloth, $5.00.)

Ziegler's pathology has become such a classic that it requires no introduction to the teachers or students in America. It has been an accepted authority in this department for nearly a generation. The tenth edition of the work just published in Germany, shows a very radical revision in most of its sections. It is scarcely necessary to add that it is quite abreast of the period in every way and reflects in a remarkable degree the reputation of its distinguished author for clear, lucid and scientific treatment of the subject.

The general arrangement of the topics remains about the same as in former editions. The author does not change his opinion as to the nature and character of tumor formation, but believes his standpoint has been strengthened in spite of the attacks directed toward him. The chapters on animal and vegetable parasites have been carefully revised and enriched by numerous illustrations. The references have been brought down to 1900 and many American annotations have been added by the translator.

The translation has been exceptionally well rendered and much credit is due Professor Warthin for his painstaking work. Many new illustrations, some of which are in colors, also appear in this edition, which leaves little to be desired in the way of mechanical execution. W. C. K.

